# Variants of human DECTIN-1 rs16910526 are linked to differential reactive oxygen species production and susceptibility to tuberculosis

**DOI:** 10.1186/s12929-024-01067-w

**Published:** 2024-08-05

**Authors:** Mónica Cufré, Mercedes Pastorini, Ignacio Martín, Rodrigo Failde, Domingo Palmero, Mercedes Alemán

**Affiliations:** 1 Hospital de Infecciosas Francisco Javier Muñiz, Buenos Aires, Argentina; 2 Instituto de Medicina Experimental, IMEX-CONICET-ANM, Pacheco de Melo 3081, 1425 Buenos Aires, Argentina

**Keywords:** DECTIN-1, Tuberculosis, Reactive oxygen species

## Abstract

**Background:**

Dectin-1 is a transmembrane receptor that plays a pivotal role in recognising fungi and *Mycobacterium tuberculosis (Mtb)*. A specific variant, DECTIN-1 rs16910526, results in a truncated receptor that disrupts membrane expression and ligand binding and is clinically associated with recurrent cutaneous mycoses. Previous research has clarified the role of Dectin-1 in boosting immune defenses against mycobacteria by enhancing reactive oxygen species (ROS) production in neutrophils (PMNs). Here, we investigated the association between the rs16910526 variant and Dectin-1 expression in PMNs, as well as intracellular ROS production in response to *Mtb*. Furthermore, we explored the potential link between the rs16910526 gene variant and TB outcomes in Argentina.

**Methods:**

DNA was extracted from blood samples obtained from a cohort of 178 TB patients and healthy subjects (HS) in Argentina. PCR amplification and sequencing were performed to identify the rs16910526 variant. Flow cytometry was utilised to assess Dectin-1 expression on the PMN plasma membrane and to measure intracellular ROS levels, as indicated by the oxidation of DHR123 in response to the *Mtb* antigen.

**Results:**

PMNs carrying the rs16910526 variant exhibited diminished Dectin-1 expression and ROS production in response to *Mtb* (p < 0.0001). In a case‒control study, the rs16910526 variant had an allelic frequency of 0.112 in TB patients and 0.051 in HS. Notably, 10 out of 88 HS and 18 out of 62 TB patients harboured the variant (odds ratio [OR]: 2.55 [95% CI 1.1–5.9, p = 0.03]), indicating a potential association with TB disease. Furthermore, TB patients with the rs16910526 variant exhibited a delayed sputum smear conversion time (p < 0.004) and 100% positivity for acid-fast bacilli smears (p < 0.00001).

**Conclusion:**

Our study identified a significant association between the SNP variant rs16910526 in the DECTIN-1 gene and Dectin-1 expression in the PMN, leading to altered ROS production. The higher frequency of this variant in TB patients compared to HS suggests a possible link with susceptibility to TB disease in Argentina.

**Graphical Abstract:**

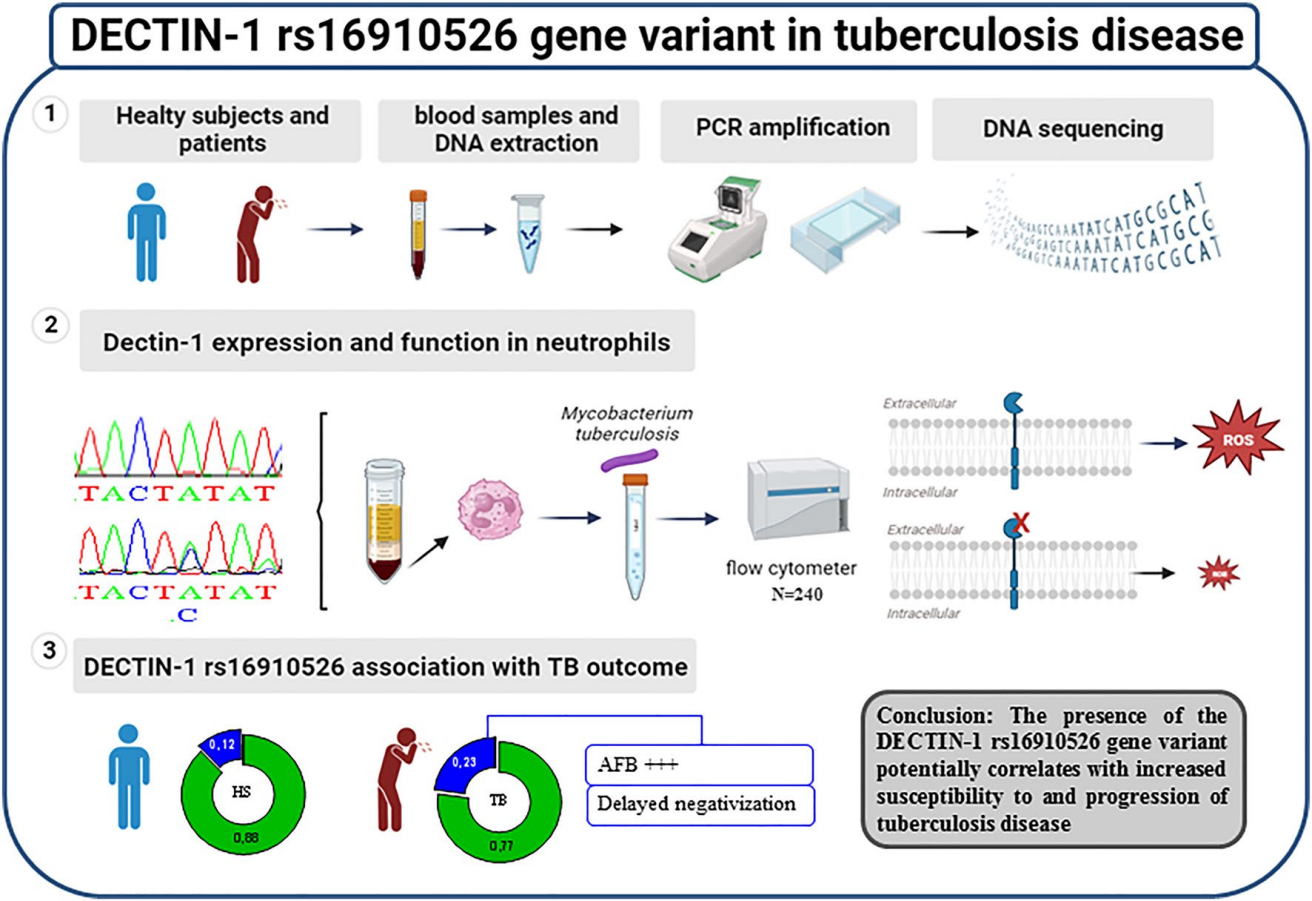

## Introduction

*Mycobacterium tuberculosis (Mtb)* is a global pathogen infecting approximately 2 billion people, leading to about 10 million new active tuberculosis (TB) cases and causing around 1.6 million deaths annually as of 2021 [1]. Despite Mtb's relatively low penetration rate of approximately 25%, only a small fraction of infected individuals progress to active disease [2, 3]. The onset of TB is influenced by complex interactions between *Mtb*, environmental factors, and host genetics. Variations in TB incidence among different races, ethnicities, and families highlight a genetic predisposition to susceptibility [4]. Genetic variations in molecules associated with innate host defence mechanisms correlate with host susceptibility to TB. In particular, numerous single nucleotide polymorphisms (SNPs) have been identified as potential factors contributing to TB resistance or susceptibility. Studies exploring SNPs in TB have pinpointed genetic differences in Toll-like receptor 2 (TLR2) genes, affecting susceptibility to TB. This finding is crucial because TLR2 recognises *Mtb* and its cell wall components, making it one of the most prevalent genetic variants associated with TB [5]. However, within this context, Dectin-1 (dendritic cell-associated C-type lectin-1) is the only pattern recognition receptor of *Mtb* for which genetic variants have not yet been thoroughly explored in the context of TB disease.

Dectin-1 is a transmembrane receptor belonging to the glycosylated C-type lectin family (CLR). It consists of an extracellular C-type lectin-like domain (CRD) and a cytoplasmic domain resembling an immunoreceptor tyrosine-based activation motif (ITAM). This receptor initiates downstream signalling and cellular activation and is encoded by the CLEC7A gene, which spans more than 10 million base pairs (Mbps) on human chromosome 12, encompassing six exons [6]. Dectin-1, which is primarily found on monocytes, macrophages, dendritic cells (DCs), neutrophils (PMNs), and a specific subset of T cells, has been extensively researched as a pivotal receptor for fungal β-1, 3-glucans. Dectin-1 can elicit diverse immune responses, including the generation of reactive oxygen species (ROS) [7]. While traditionally studied for its role in defending against fungal infections [8–10], research suggests that this receptor recognises diverse ligands beyond β-glucans [11]. This interaction occurs concurrently with TLR2 and has significant immunological implications, including cytokine secretion [12–14]. Our previous studies have described how *Mtb* simultaneously interacts with Dectin-1 and TLR2 to induce ROS generation. This process triggers the activation of p38 MAPK and Syk, initiating programmed cell death in PMNs, which aids in controlling lung tissue inflammation. Moreover, it enhances antigen presentation, contributing to the immune response against TB [15, 16].

A genetic variant of the Dectin-1 gene involves an early stop codon mutation consisting of a SNP (rs16910526, exon 6, chromosome 12; location, 10162354 bp). The mutation (A → C) causes a change in amino acid 238 from tyrosine to a stop codon (Tyr238X), leading to the loss of the last nine amino acids of the CRD. Consequently, this truncation reduces its expression on the cell surface as well as its capacity to bind β-glucan [17, 18]. This genetic variation has been linked to onychomycosis, recurrent vulvovaginal candidiasis, or both [19] and affects the secretion of proinflammatory cytokines [19, 20].

Having established the significant role of Dectin-1 in generating *Mtb*-induced ROS, our primary objective was to investigate the potential correlation between the presence of the rs16910526 SNP and an impaired ability to initiate the respiratory burst in immune cells responding to *Mtb* antigens. In pursuit of this objective we aim to gain a comprehensive understanding of how this genetic variant might affect the innate immune response against TB. This exploration seeks to provide valuable insights that could substantially advance both diagnostic and treatment approaches for this disease.

## Materials and methods

### TB patients and healthy subjects (HS)

All subjects were recruited between 2017 and 2020, were vaccinated with Bacille Calmette-Guérin (BCG) at birth, had no underlying diseases such as cancer, diabetes, or immune-related conditions, and were HIV-negative. The TB patients were diagnosed with active TB at Hospital Muñiz in Buenos Aires, Argentina, which receives the highest volume of TB suspicion from various sources. Diagnosis involved clinical and radiological assessments, acid-fast bacilli detection in sputum samples, and isolation of *Mtb* in culture. The HS group comprised household contacts and healthy donors from Fundación Hemocentro Buenos Aires. All HS lacked a history of TB (confirmed through chest X-rays and analysis of acid-fast bacilli in sputum) and tested negative for the QuantiFERON-TB Gold In-Tube test (QFT-GIT, Qiagen, USA). Peripheral blood was collected in heparinised tubes from all participants over 18 years old after provided written informed consent for sample collection and analysis. The research procedure complied with the principles outlined in the Declaration of Helsinki and was approved by the Ethical Committee of Muñiz Hospital (protocol code 988/18) and the Ethics Committee of the National Academy of Medicine in Buenos Aires.

### Genetic screening for DECTIN-1 rs16910526 variant

Genomic DNA was extracted from whole blood samples from 80 TB patients and 98 HS using Puro Genomic DNA Extraction Kits (PB-L, Productos Bio-Logicos^®^, Argentina). The presence of the rs16910526 SNP in the DECTIN-1 gene was investigated via polymerase chain reaction (PCR), which amplified the specific DECTIN-1 sequence. Primers targeting the region of interest were used for amplification, sourced from GenBank (chromosome position 12p13, NC-000012.10). The sequences of the primers used are: Sense: 5'AATCACAGCCTCTCCCTTCA 3', Reverse: 5'GATTTAAGCCTCCTTTTCCAA 3'. Subsequent sequencing of the exonic and neighbouring intronic regions of the DECTIN-1 gene was carried out. High-fidelity DNA polymerase and dNTPs (PB-L) were used for PCR, which included 35 cycles of amplification at 94 °C for 5 min, 94 °C for 30 s, 58 °C for 50 s, and 72 °C for 45 s per cycle. For quality control purposes, each PCR set comprised randomly chosen replicates of samples previously typed in the study, along with two negative controls. Consistency in quality control and duplicate samples reached or exceeded 99% agreement across all assays. Importantly, laboratory personnel remained unaware of the sample statuses throughout the process, and sequencing procedures were conducted at Macrogen after amplification.

### Antigens

Gamma-irradiated H37Rv *Mtb* strain was acquired from Bei Resources, NIAID, NIH (Bethesda, MD, USA). Gamma-irradiated bacteria are inactive and non-infectious without losing the structure of the cell wall; the absence of replication of the irradiated bacteria was verified at the mycobacteria service of ANLIS-Malbrán. The bacteria were finally suspended in phosphate-buffered saline (PBS) at an optical density of 600 nm of 1 (~ 10^8^ bacteria/mL). The suspension was stored at −20 °C until experimentation.

### Cell preparation and culture

PMNs were obtained from heparinised venous blood using Ficoll-Hypaque gradient centrifugation for 40 min at 350 g and 4 °C, followed by sedimentation for 20 min in a 3% dextran solution (Sigma Chemical Co., St. Louis, Mo, USA). Red blood cells were removed through hypotonic lysis, and PMNs were washed and suspended in RPMI-1640 medium supplemented with 1% heat-inactivated fetal calf serum (FCS), 100 U/mL penicillin, and 100 μg/mL streptomycin (Gibco, NY, USA) at a concentration of 3 × 10^6^ cells/mL. The cell viability exceeded 95%, as determined by trypan blue dye exclusion, and the purity reached 95%, as validated through Wright‒Giemsa staining and fluorescence-activated cell sorting (FACS) light scatter pattern analysis.

### Oxidative bursts assay

Intracellular reactive oxygen species (ROS) were measured using the 123Dihydrorhodamine assay (DHR), a dye that freely enters the cell membrane and, after oxidation by ROS to rhodamine 123, it emits a bright fluorescent signal. Briefly, 5 × 10^5^ PMNs were incubated with 100 μl of DHR solution (5 μg/mL) at 37 °C for 15 min. Then, *Mtb* (1 × 10^6^ bacteria/mL) was added for 90 min alongside a positive control of phorbol myristate acetate (PMA) (10 ng/mL) (Sigma) and a negative control of medium alone. Afterward, the PMNs were washed, and 10.000 events were collected using flow cytometry (BD Biosciences) with FSC, SSC, and FL-1 parameters. The results are presented as the index of ROS calculated as the mean fluorescence intensity (MFI) after *Mtb* stimulation / MFI after culturing with medium.

### Dectin-1 expression

Freshly isolated PMNs were assessed using saturating concentrations of monoclonal mouse anti-human Dectin-1-PE-conjugated antibodies (clone 15E2), along with their corresponding isotype controls (R&D Systems Inc., Minneapolis, USA). Briefly, 5 × 10^5^ cells were incubated for 20 min on ice, washed, and fixed with 500 μL of 1% paraformaldehyde. Subsequently, 10.000 cells were collected in a flow cytometer and analysed as described above. The results are presented as the mean fluorescence intensity (MFI).

### Statistics

The genotype and allele frequencies were determined through direct counting. Hardy–Weinberg (HW) equilibrium was individually tested for cases and controls using the χ2 test. Genotype frequencies were compared using Fisher’s exact test. Odds ratios (ORs) with 95% confidence interval (CI) were calculated to estimate genotype associations. Correlation analysis was performed using Spearman’s test. Quantitative data are presented as the mean ± standard error of the mean (SEM). Differences among groups were assessed using the Mann–Whitney U test for unpaired and nonparametric samples. Statistical analyses were conducted using GraphPad Prism v7.0 or R software. A p value less than 0.05 was considered to indicate statistical significance.

## Results

### Demographic and haematological characteristics of the population studied

Laboratory assessments at diagnosis revealed an increased total white blood cell count in TB patients, as previously documented [21] (Table 1), which correlated with an increased percentage of PMNs, a characteristic trait associated with the severity and extent of symptoms [22]. Although no differences were detected regarding the distribution of ages, notable disparities emerged concerning ethnic origins (p < 0.0001) and gender composition (p < 0.04) within both populations. However, no differences in genotype distributions were found by sex within each population (data not shown).

**Table 1:** Demographic characteristics and laboratory findings at diagnosis in healthy subjects (HS) and TB patients

N	HS 120	TB 120	*P* value HS vs TB
Age (Mean; ± SEM)	30,2 ± 7,8	29,6 ± 9,5	0.103^a^
Leukocytes × 10^3^/mm^3^	7,2 ± 1,3	12,1 ± 2,7	< 0.002^a^
PMN %	65,2 ± 6,9	79,0 ± 6,1	< 0.002^a^
Lymphocytes %	31,8 ± 1,1	25,5 ± 4,7	0.12^a^
Monocytes %	5,3 ± 0,2	4,2 ± 0,6	0.08^a^
Ethnicity (%)			
White individuals	72 (60)	45 (37.5)	< 0.0001^b^
Native American individuals	48 (40)	75 (62.5)	
Sex (%)			
Male	65 (54.2)	78 (65)	< 0.04^b^
Female	55 (45.8)	42 (35)	

Categorical variables are expressed as percentages. Age values are presented as the mean ± standard error of the mean (SEM).

^a^*p* values were calculated using the Mann‒Whitney U test for unpaired samples. ^b^*p* values were calculated using Fisher’s exact test for categorical variables. *N* number of individuals

### Evaluation of ROS generation and Dectin-1 expression in PMNs from HS and TB patients

**Fig. 1:**
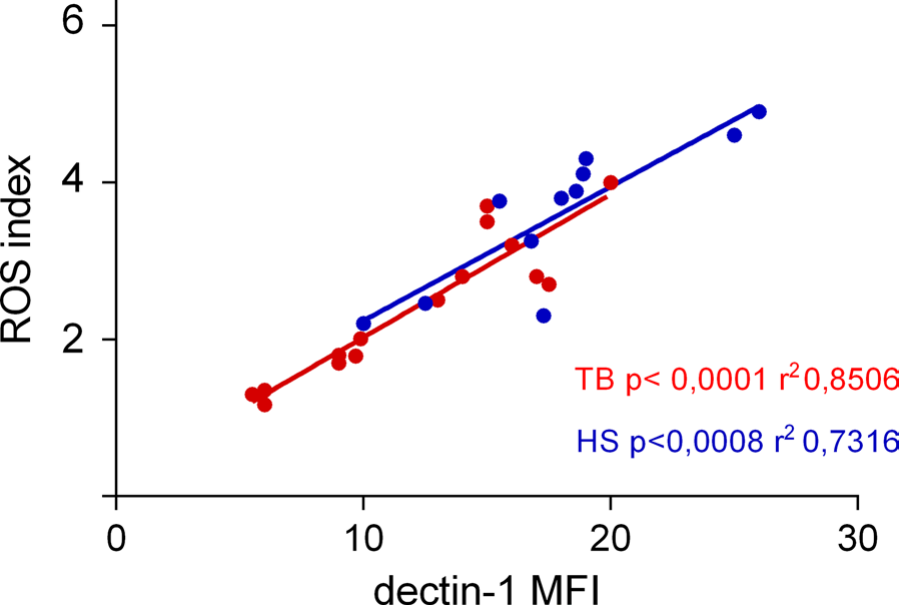
Correlation between Dectin-1 expression and ROS index in response to *Mtb* in PMM from HS and TB patients. PMNs were incubated with DHR and treated with *Mtb* or left unstimulated for 90 min. Oxidized DHR emission due to ROS generated in the presence of *Mtb* was then assessed using flow cytometry in the FL-1 channel. The ROS index was calculated as the mean fluorescence intensity (MFI) after *Mtb* stimulation / MFI after culturing with medium. Dectin-1 expression in fresh peripheral blood PMNs was assessed by labelling with anti-Dectin-1-PE antibodies and the corresponding nonspecific isotype (IgG) using flow cytometry. HS: p < 0.0008, r2 = 0.7316; TB p < 0.0001, r2 = 0.8506 (Spearman test)

The generation of ROS is crucial for eliminating invasive pathogens, promoting apoptosis in PMNs and reducing inflammation by eliminating infected PMNs from the infection site [23, 24]. A previous study revealed that non-opsonized *Mtb*-induced ROS production hinges on the interaction of *Mtb* with both Dectin-1 and TLR2, leading to PMN apoptosis without affecting Dectin-1 expression over time [25]. Using DHR dye, we detected greater ROS levels in the PMNs from the HS group than in those from the TB group (Table 2), in response to *Mtb*. Although Dectin-1 expression tended to be lower in TB patients, the differences were not significant. Nonetheless, we noted a positive correlation between ROS production and Dectin-1 expression (Fig. 1).

**Table 2:** ROS generation and Dectin-1 expression in PMNs from HS and TB patients

	HS	TB	*P* value HS vs TB
ROS (MFI)	712,7 ± 26,03	512,1 ± 25,43	0.0001^a^
ROS (index)	3,8 ± 0,23	2,7 ± 0,26	0.0001^a^
Dectin-1 (MFI)	22,3 ± 1,4	14,5 ± 1,1	0.051

ROS (MFI), Mean Fluorescence Intensity of Oxidized DHR emission by flow cytometry in the FL-1 channel. ROS index, ROS generated in the presence of *Mtb* compared to that of the controls. Dectin-1 (MFI), Mean Fluorescence Intensity of Dectin-1 expression in fresh peripheral blood PMNs assessed by labelling with anti-Dectin-1-PE antibodies using flow cytometryThe values are expressed as the mean ± standard deviation (SEM).

^a^*P* values were calculated using the Mann–Whitney U test for unpaired and nonparametric samples

### Distribution of genotype and allele frequencies of the rs16910526 SNP in HS and TB patients

In a case–control study, differences in the allelic distribution and genotype frequencies of the rs16910526 SNP were significant between TB patients and HS. The C allele was notably more prevalent among TB patients (TB = 11.2% and HS = 5.1%, p = 0.042), suggesting an overrepresentation in TB individuals. Genotype frequencies also differed significantly (p = 0.037), particularly with the AC genotype being more common in TB patients (TB = 23% and HS = 12%). Logistic regression analysis showed an odds ratio of 2.55 (95% CI 1.1–5.9, p < 0.03), indicating increased susceptibility to TB for individuals carrying the AC genotype compared to those with the AA genotype (Table 3). Both populations were in Hardy‒Weinberg equilibrium for this SNP (p = 0.77473). Notably, no individuals with the homozygous genotype (CC) were found in the total population studied.

**Table 3:** Distribution of genotypes and allele frequencies of the rs16910526 SNP variant among patients (TB) and healthy subjects (HS)

	TB # (%)	HS # (%)	*p* value TB vs HS	OR (95% CI)/*p* value
Genotypes^a^N = 178				
AC	18 (23)	10 (12)	0.037^b^	2.55 (1.1–5,9)/ < 0,03^b^
AA	62 (77)	88 (89.8)		
Alleles N = 356				
C	18 (11.2)	10 (5.1)	0.046^b^	2.36 (1.06–5.26)/ < 0,04^b^
A	142 (88.8)	186 (94.9)		

^a^Dominant model AC vs AA. ^b^*P* values were calculated by Fisher’s exact test. OR, odds ratio risk analysis. The genotypes were in Hardy‒Weinberg equilibrium (Ho = 0; observed – expected by HW): chi-square (χ2) test ns (p = 0.77473)

### Association of rs16910526 SNP genotypic variants with ROS production and Dectin-1 expression

**Fig. 2:**
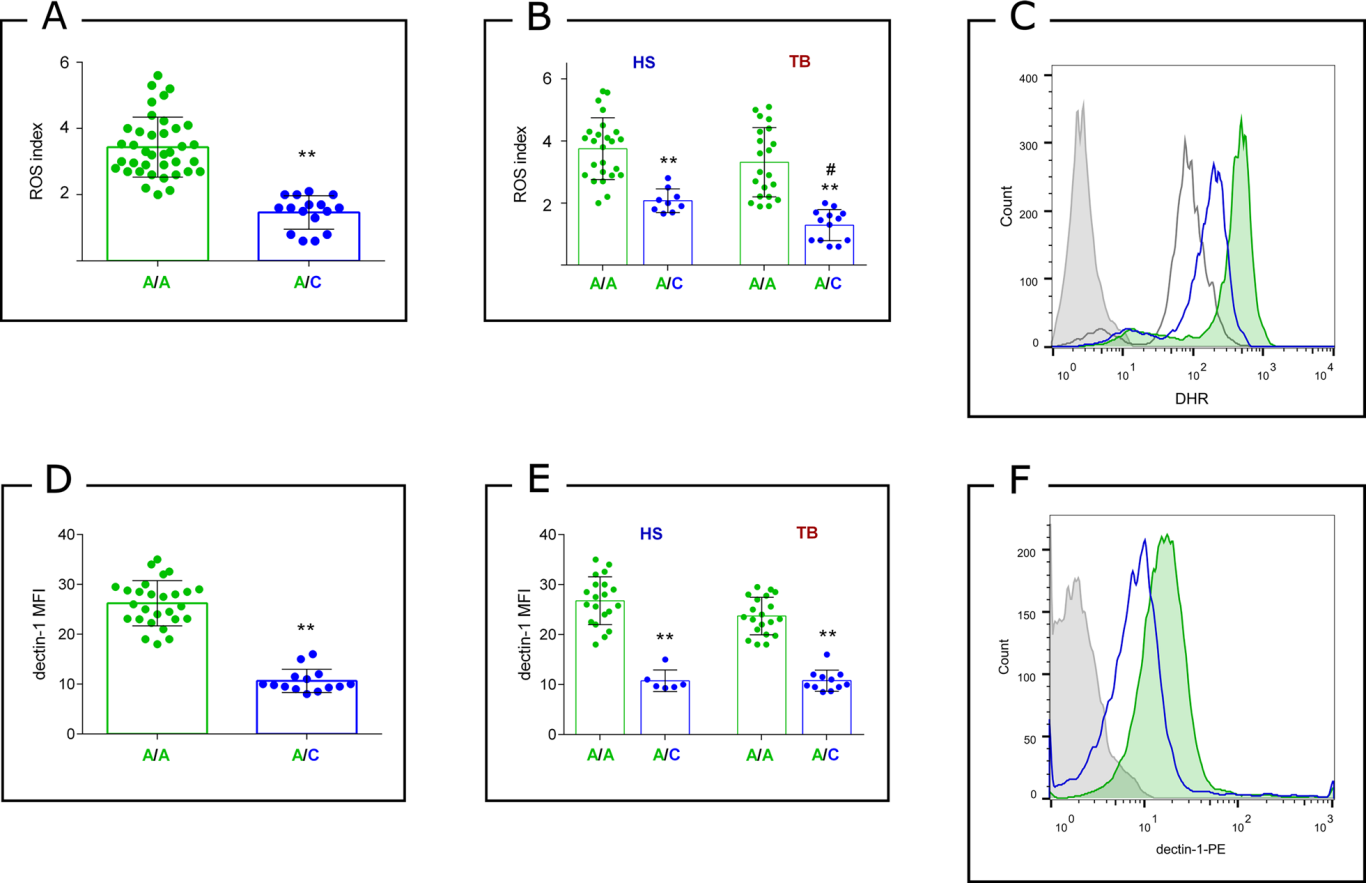
*Mtb*-induced ROS production and Dectin-1 expression in PMNs harbouring rs16910526 genotypic variants. **A** ROS index in the total population (**p < 0.0001); **B** ROS index in HS and TB patients (A/A versus A/C genotypes **p < 0.0001; A/C in HS vs. TB #p < 0.02); **C** Representative histogram of DHR emission in samples with A/A and A/C genotypes compared to control (grey line) and unlabelled cells (grey shadow); **D** Dectin-1 expression in the PMN in the total population (A/A versus A/C genotypes **p < 0.0001); **E** Dectin-1 expression in HS and TB patients (A/A versus A/C genotypes **p < 0.0001); **F** A representative histogram of Dectin-1 expression in two different individuals (A/A and A/C**)** and the corresponding nonspecific isotype (IgG) labelling (grey shadow). The values are expressed as the mean ± SEM of the MFI determined by flow cytometry. P values were calculated using the Mann–Whitney U test for unpaired and non-parametric samples

Subsequently, we investigated the increase of ROS induced by *Mtb* in PMNs of different genotypes. Significantly lower ROS production were observed in PMNs with the A/C genotype compared to those with the A/A genotype following *Mtb* stimulation (p < 0.0001) (Fig. 2A). When analysing TB patients and HS separately, we found that in both cohorts, the presence of the C allele was associated with reduced ROS production (Fig. 2B). Interestingly, while the ROS index was similar in homozygotes from both cohorts, TB patients with the A/C genotype showed lower ROS induction than HS individuals with the same genotype (p < 0.02), suggesting that factors beyond Dectin-1, such as other unexamined polymorphisms, may contribute to this difference.

Furthermore, we evaluated the expression of Dectin-1 in PMNs across different genotypes. Consistent with previous reports (20), Dectin-1 expression was significantly lower in PMNs with the A/C genotype compared to those with the A/A genotype (Fig. 2D–F). Notably, individuals without the genetic variant showed no differences in the expression of Dectin-1 between HS and TB patients (Fig. 2E).

### Associations between rs16910526 SNP genotypic variants and clinical parameters

In our subsequent analysis, we explored associations between the rs16910526 variant and TB severity by examining clinical parameters. The white blood cell count and PMN percentage differed between the TB group and the HS group (Table 1), but there were no differences in the distribution of the rs16910526 variant (Table 4). Symptoms upon admission in TB patients, including weight loss, night sweats, discomfort or weakness, persistent fever, cough, history of respiratory difficulty, and hemoptysis, displayed no notable differences between genotypes except for AFB in sputum smears (p < 0.00001) or sputum conversion duration (p < 0.004) suggesting that the variant may hinder pathogen control.

**Table 4:** Associations between clinical parameters and the rs16910526 SNP genotypic variant during active TB

TB patients n = 53	rs16910526	Genotype	*P* value
	AA	AC	
Leucocytes × 10^3^/mm^3^	9,8 ± 1,1	9,6 ± 1,1	0,88^a^
PMN (%)	75 ± 3,0	71 ± 5	0.92^a^
AFB—o AFB +	30 (75%)	0 (0%)	< 0.00001^b^
AFB + + +	10 (25%)	13 (100%)	
Negativization (days)	19,5 ± 2,0	33,2 ± 3,6	< 0,004^a^

Leukocyte count and PMN percentage in peripheral blood. Acid-fast bacilli in sputum smears are classified as follows: AFB−: absence of bacilli; AFB + : 1–9 bacilli/100 fields; AFB +  + : 1–9 bacilli/10 fields; AFB +  +  + : 1–9 bacilli/field. Negativization: the period for a sputum sample to show an absence of bacilli (AFB−) in days. Continuous data are presented as the mean ± standard error of the mean (SEM), and categorical data are presented as numbers (genotype percentages)

^a^*p* values were calculated by the Mann–Whitney U test for unpaired and nonparametric samples. ^b^*P* values were calculated by Fisher’s exact test

## Discussion

Certain genetic variants may influence the functionality of the immune system, thereby impacting its ability to recognise pathogens [5]. Although the significant role of Dectin-1 in regulating the immune response against *Mtb* has been extensively documented [14, 26, 27], the genetic variants of Dectin-1 in TB remain unaddressed. Avoiding ROS production significantly affects the survival of both bacteria and PMNs, potentially assisting *Mtb* in evading the host's immune defenses, as observed in prevalent *Mtb* strains in Argentina [28, 29]. This strategy mirrors observations in other pathogens, such as *Anaplasma phagocytophilum*, which, by failing to induce ROS production, delays PMN apoptosis [30]. The importance of ROS generation in the immune response against *Mtb* is underscored by the fact that chronic granulomatous disease (CGD) is the primary immunodeficiency most clearly associated with susceptibility to TB [31]. Thus, epidemiological outcomes are shaped by the genetic background of the host and pathogen, their interactions, and the surrounding environment. We chose the rs16910526 SNP due to its significance, despite the identification of five other SNPs located in the 3′UTR (rs11053593 and rs7959451) and intronic region (rs7309123, rs2078178, and rs3901533), which have been clinically linked to various diseases [32–35]. However, unlike the rs16910526 SNP, there is no definitive evidence of their functional implications.

In this study, we provide evidence that the presence of the rs16910526 genetic variant correlates with reduced Dectin-1 expression on the PMN membrane, consistent with findings in monocytes reported by other researchers [19, 20, 36]. This decrease in Dectin-1 expression translates to a diminished capacity for ROS generation in response to *Mtb*. Notably, the decreased *Mtb*-induced ROS production in the PMNs of TB patients appears to be specifically associated with Dectin-1, as previous investigations have indicated heightened activation and increased ROS levels in response to fMLP, a nonspecific chemoattractant stimulus [22]. Therefore, the reduced ROS production in response to *Mtb* cannot be attributed to an intrinsic characteristic of PMNs from TB patients [37]. Furthermore, individuals carrying the allelic variant also exhibit a diminished capacity for ROS generation in their monocytes (data not shown). However, it is important to consider the potential presence of certain SNPs in TLR2 that may further reduce the capacity to generate ROS in TB patients. Additionally, this decrease should not be attributed to a lower Dectin-1 protein level in TB patients, as no differences were observed between HS and TB patients who did not carry the allelic variant (Fig. 2B). In this context, mRNA expression was found to be equivalent among the different genotypes [20], indicating the absence of Dectin-1 protein expression on the membrane of cells from individuals heterozygous for the genetic variant. Further analysis of the Dectin-1 protein structure bearing an early stop codon revealed the absence of a crucial cysteine disulfide bridge, likely resulting in significant functional implications, as well as the absence of cell membrane expression of Dectin-1 [19].

Neutrophils, the primary cells that rapidly mobilise to infection sites, are commonly recognised for their localised functions, with limited evidence of returning to the bloodstream. The continual influx of activated PMNs from the bloodstream, as indicated by the secretion of IL-1, TNF-α, and IL-8 [22], is correlated with an increased presence of PMNs in the BALF of individuals with active TB [38]. These observations are closely linked to disease severity and the intensity of the inflammatory response [39]. Our study revealed no correlation between the rs16910526 gene variant and white blood cell counts or PMN counts in TB patients. However, this variant was associated with increased AFB levels in sputum smears and prolonged sputum conversion duration, suggesting that this variant may impede pathogen control, likely through ROS signalling, which is vital for innate and adaptive immune responses. Nevertheless we did not explore a direct association between ROS reduction and TB susceptibility, which remains to be established.

Our findings, revealed an overrepresentation of the rs16910526 SNP variant in TB patients, suggesting a potential association with TB susceptibility within the analysed population. However, Dectin-1-deficient mice are resistant to *Mtb* infection, similar to wild-type mice, indicating that Dectin-1 may play a redundant role in antimycobacterial defense [40].

The intricate nature of TB transmission across diverse populations involves several factors beyond genetic susceptibility. Overcrowded living conditions, inadequate nutrition, weakened immunity, and poor ventilation [41] heighten infection risks [42] and hinder treatment effectiveness. Individuals affected by TB often live in substandard conditions, leading to delays in medical care, especially among those experiencing persistent cough or fever. The study cohort included homeless individuals and, occasionally, nearby immigrants who frequently work in cramped conditions, potentially facilitating disease spread [43]. Although no scientific evidence supports the transmission of Andean strains to Argentinean patients, the dominant strains in Buenos Aires spread among immigrants contracting the disease in clandestine textile workshops. These contextual elements clarify demographic disparities, not solely due to the higher frequency of genetic mutations. Instead, it signifies a complex interplay among social, environmental, and occupational factors influencing disease spread among diverse groups. In this context, one of the main limitations in this study was the difficulty in assembling a larger cohort of healthy household contacts of the patients, as well as the relatively small size of the recruited cohort. The onset of the COVID-19 pandemic may have influenced the immunological status of both healthy individuals and TB patients, prompting us to stop recruiting people. Nonetheless, it is worth noting that the allelic frequency of this variant was 0.09, which is slightly above the global average [19].

## Conclusion

Our study identified a significant association between the SNP variant rs16910526 in the DECTIN-1 gene and the expression of Dectin-1, leading to impaired ROS production. Moreover, we observed a potential link between susceptibility to TB and the SNP rs16910526 in the Argentine population. More research is needed to elucidate whether or not there is a direct relationship between the respiratory burst and the development of TB disease. Additionally, investigating the combined effects of TLR2 genetic variants is warranted, given the collaborative role of TLR2 and Dectin-1 in recognising *Mtb*.

## Data Availability

All data generated or analysed during this study are included in this published article.

## Abbreviations

AFB: Acid–Fast Bacilli Smear
BALF: Bronchoalveolar lavage fluid
CLR: C-type lectin family
Dectin-1: Dendritic Cell-associated C-type lectin-1
DHR: 123-Dihydrorhodamine
fMLP: N-formyl-methionyl-leucyl-phenylalanine
IL-1: Interleukin-1
IL-8: Interleukin-8
MFI: Mean fluorescence intensity
*Mtb*: *Mycobacterium tuberculosis*
PBS: Phosphate-buffered saline
PCR: Polymerase chain reaction
PMA: Phorbol Myristate Acetate
PMN: Polymorphonuclear Neutrophil
ROS: Reactive oxygen species
SNP: Single nucleotide polymorphism
TB: Tuberculosis
TLR: Toll-like receptor
TNF-α: Tumour Necrosis Factor-alpha
